# Rehospitalization following percutaneous coronary intervention for commercially insured patients with acute coronary syndrome: a retrospective analysis

**DOI:** 10.1186/1756-0500-5-342

**Published:** 2012-07-02

**Authors:** Eric S Meadows, Jay P Bae, Anthony Zagar, Tomoko Sugihara, Krishnan Ramaswamy, Rebecca McCracken, Darell Heiselman

**Affiliations:** 1 Eli Lilly and Company, Indianapolis, IN, USA; 2 inVentiv Clinical Solutions, LLC, Indianapolis, IN, USA; 3 Daiichi Sankyo Inc, Parsippany, NJ, USA; 4 i3 Statprobe, Minneapolis, MN, USA; 5 Global Health Outcomes, Eli Lilly and Company, Indianapolis, IN, 46285, USA; 6 Present address: MedMining, Geisinger Health System, Danville, PA, 17822, USA

## Abstract

**Background:**

While prior research has provided important information about readmission rates following percutaneous coronary intervention, reports regarding charges and length of stay for readmission beyond 30 days post-discharge for patients in a large cohort are limited. The objective of this study was to characterize the rehospitalization of patients with acute coronary syndrome receiving percutaneous coronary intervention in a U.S. health benefit plan.

**Methods:**

This study retrospectively analyzed administrative claims data from a large US managed care plan at index hospitalization, 30-days, and 31-days to 15-months rehospitalization. A valid Diagnosis Related Group code (version 24) associated with a PCI claim (codes 00.66, 36.0X, 929.73, 929.75, 929.78–929.82, 929.84, 929.95/6, and G0290/1) was required to be included in the study. Patients were also required to have an ACS diagnosis on the day of admission or within 30 days prior to the index PCI. ACS diagnoses were classified by the International Statistical Classification of Disease 9 (ICD-9-CM) codes 410.xx or 411.11. Patients with a history of transient ischemic attack or stroke were excluded from the study because of the focus only on ACS-PCI patients. A clopidogrel prescription claim was required within 60 days after hospitalization.

**Results:**

Of the 6,687 ACS-PCI patients included in the study, 5,174 (77.4%) were male, 5,587 (83.6%) were <65 years old, 4,821 (72.1%) had hypertension, 5,176 (77.4%) had hyperlipidemia, and 1,777 (26.6%) had diabetes. At index hospitalization drug-eluting stents were the most frequently used: 5,534 (82.8%). Of the 4,384 patients who completed the 15-month follow-up, a total of 1,367 (31.2%) patients were rehospitalized for cardiovascular (CV)-related events, of which 811 (59.3%) were revascularization procedures: 13 (1.0%) for coronary artery bypass graft and 798 (58.4%) for PCI. In general, rehospitalizations associated with revascularization procedures cost more than other CV-related rehospitalizations. Patients rehospitalized for revascularization procedures had the shortest median time from post-index PCI to rehospitalization when compared to the patients who were rehospitalized for other CV-related events.

**Conclusions:**

For ACS patients who underwent PCI, revascularization procedures represented a large portion of rehospitalizations. Revascularization procedures appear to be the most frequent, most costly, and earliest cause for rehospitalization after ACS-PCI.

## Background

Acute coronary syndrome (ACS) includes ST-segment elevation myocardial infarction (STEMI), non-ST-segment elevation myocardial infarction (NSTEMI), and unstable angina (UA). Approximately 733,000 patients discharged from the hospital in 2006 had a primary diagnosis of ACS [1]. ACS can lead to both mortality and morbidity during and after hospitalization, with up to 30% of discharged patients needing rehospitalization within 6 months [2-4]. ACS management includes treating evolving acute STEMI, and preventing the progression of UA and NSTEMI into acute STEMI and death, by hospitalization and the use of antiplatelet and anticoagulant therapy, either alone or combined with early revascularization [2,5]. Percutaneous coronary intervention (PCI) is generally recommended for patients with either STEMI or NSTEMI/UA. PCI represents medical procedures (such as bare-metal stents [BMS] or drug-eluting stents [DES] and balloon angioplasty) that use “mechanical” means to treat patients with partially or completely restricted blood flow through an artery of the heart [6]. Only 25% of hospitals in the United States have the equipment, expertise, and facilities to administer PCI, and these hospitals are referred to as PCI-capable hospitals [6]. An estimated 1,313,000 PCI procedures were performed in the United States in 2006; approximately 65% on men and approximately 50% were performed on patients ≥65 years old [1]. In 2006, approximately 76% of stents used during PCI were DES with the remaining 24% being BMS [1].

Hospital readmission rates following PCI are an important measure of quality of care and also have important economic implications for the overall healthcare system. In general, the National Quality Forum has adopted the rate of rehospitalization as an important measure of hospital quality and the Centers for Medicare and Medicaid Services (CMS) has recommended that rehospitalization rates be incorporated as a measure for value-based hospital reimbursement [7,8].

While prior research has provided important information about readmission rates following PCI, reports regarding charges and length of stay (LOS) for readmission beyond 30 days post-discharge for ACS-PCI patients in a large cohort are limited. Previous research investigating rehospitalization has focused on specific subpopulations (such as Medicare fee-for-service), 30-day and 1-year rehospitalization, predictors of rehospitalization, and total costs over a 1-year period [9-11]. The primary objective research questions of the current study were from the managed care perspective and included the following: What is the rate of rehospitalization for commercially-insured ACS-PCI patients at 30 days post-index PCI? What is the rehospitalization rate within 15 months? What procedures and diagnoses were associated with these rehospitalizations? What was the LOS and charges associated with these rehospitalizations?

## Methods

This retrospective database analysis used administrative claims data to characterize ACS-PCI patients in a large US managed care plan at index hospitalization, 30-days, and 31-days to 15-months rehospitalization. Data were analyzed in a manner compliant with the Health Insurance Portability and Accountability Act, and no identifiable protected health information was extracted. This research study involved analysis of pre-existing, de-identified data and was therefore not reviewed by an Internal Review Board.

**Figure 1:**
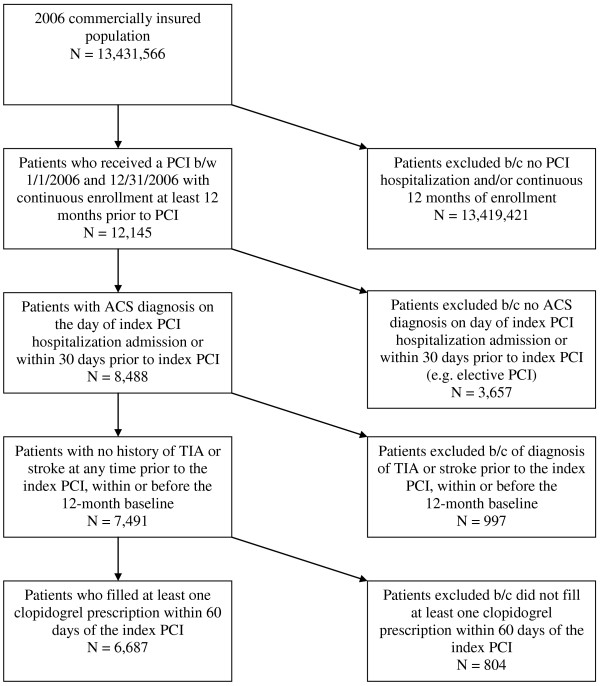
** Flow diagram of sample selection.** Abbreviations: ACS = acute coronary syndrome; b/c = because; b/w = between; PCI = percutaneous coronary intervention; TIA = transient ischemic attack.

The study cohort included commercially insured patients, with both medical and pharmacy benefits, who received a PCI between January 1, 2006 and December 31, 2006. Figure 1 presents the study’s sample selection process. A valid Diagnosis Related Group (DRG) code (version 24) associated with a PCI claim was required for patients to be included in the study. In addition, continuous enrollment in a health plan was required for at least 12 months prior to the PCI, which was defined as the baseline period. No minimum continuous enrollment was required post-PCI. For patients with more than one PCI procedure, the first PCI in 2006 was considered the index event. If more than one DRG code was identified for a single hospitalization, the highest weighted DRG code (according to CMS criteria) was assigned. Patients selected for this study were required to have an ACS diagnosis on the day of admission or within 30 days prior to the hospitalization for the index PCI procedure. The ACS diagnoses were classified by International Statistical Classification of Disease 9 (ICD-9) codes as either STEMI (410.xx other than 410.7), NSTEMI (410.7), or UA (411.1). The diagnosis and procedure codes used to identify patients are summarized in Additional file 1: Diagnosis and Procedure Codes. In order for our analyses to be applicable to both clopidogrel and prasugrel (though not commercially available in the time frame of our study), patients who had a diagnosis of transient ischemic attack or stroke at any time prior to the index PCI, within or before the 12-month baseline period, were excluded. Because clopidogrel has been the standard medical treatment for ACS-PCI, patients were also excluded if they did not have at least one prescription for clopidogrel within 60 days of the initial PCI hospital admission. Patients not receiving anti-platelet therapy after PCI have previously been shown to have different characteristics, behaviors, treatment, and outcomes [12] and were therefore outside the scope of the current study. Clopidogrel is preferred over ticlopidine and is more widely used, with ticlopidine comprising less than 0.1% of the utilization [13,14]. The 60-day grace period was selected to account for patients who could have left the hospital with a supply of clopidogrel and/or were prevalent users of clopidogrel prior to index hospitalization. Use of over-the-counter medication such as aspirin was not observable in this data source. 

Patient characteristics of interest included demographic and healthcare-related variables. Treatment patterns at index hospitalization were grouped into 4 categories based on DRG codes: PCI, coronary artery bypass graft (CABG), cardiac-related (other than PCI or CABG), and non-cardiac-related. The type of stent used (at least one DES, BMS only, or no stent) was determined from the DRG code description for the index PCI. Comorbidities such as diabetes (250.xx), hyperlipidemia (272.xx), and hypertension (401.xx through 405.xx) were determined using ICD-9 codes in the claims records for the 12-month pre-PCI baseline period. The follow-up period was 15 months beginning on the discharge date of the index hospitalization. The number of days, if any, before rehospitalization was defined as the number of days between initial discharge and readmission date.

Of the 6,687 patients in the initial hospitalization cohort, frequency distributions for first rehospitalization were dichotomized into “all-cause” and cardiovascular-related, “CV-related”. The categories were based on the DRGs assigned at rehospitalization and for CV-related the categories included stroke (DRG 014); revascularization procedures (106, 518, 555, 556, 557, 558); heart failure and shock (127); chest pain (143); and other CV (124, 125,132, 144, 515), which includes all DRGs not listed in the previous groups. CV-related readmissions were classified according to their associated DRG code. Revascularization procedures were considered to be any hospitalization with a DRG code for a PCI, stent, and/or CABG (DRGs 106, 518, 555–558).

Descriptive statistics were used to summarize the data. The LOS for index hospitalizations was defined as the maximum duration of days for all services that began on the day of admission. In the follow-up period, the LOS for services that overlapped in time was subsumed under the service that had the maximum duration of days. Inpatient medical charges were calculated as the sum of charges for all services that were associated with a hospitalization. Patients without readmissions were excluded from the calculations of rehospitalization LOS or charges. Kaplan-Meier analyses were conducted to look at time to rehospitalization for all-cause and for CV-related events. Log-rank test was used to compare the time to event for CV-related DRG rehospitalization groups.

## Results

Patient characteristics are summarized in Table 1. Of the 6,687 ACS-PCI patients included, 5,174 (77.4%) were male, 5,587 (83.6%) were <65 years old (mean age of 56.5 years), 5,204 (77.9%) were located in the South and Midwest, 4,821 (72.1%) had hypertension, 5,176 (77.4%) had hyperlipidemia, and 1,777 (26.6%) had diabetes. Drug-eluting stents were most frequently used, 5,534 (82.8%). Only 998 (14.9%) of patients were on clopidogrel therapy prior to their index PCI event.

**Table 1:** Characteristics of Study Population (N = 6687)

	**n**	**(%)**
Age		
<65	5587	(83.6)
65–74	803	(12.0)
>74	297	(4.4)
Mean ± SD	56.5 ± 9.4	
Gender		
Male	5174	(77.4)
Female	1513	(22.6)
Regions		
Midwest	2265	(33.9)
Northeast	597	(8.9)
South	2939	(44.0)
West	886	(13.2)
Index ACS Diagnoses*		
STEMI	3276	(49.0)
NSTEMI and UA	3411	(51.0)
Type of Stent at Index^†^		
Drug-eluting stent	5534	(82.8)
Bare-metal stent	787	(11.8)
No stent	62	(0.9)
Unknown	304	(4.5)
Medical History in 12-month Baseline*^†^		
Prior PCI	487	(7.3)
Prior CABG	51	(0.8)
Clopidogrel Use	998	(14.9)
Hypertension	4821	(72.1)
Hyperlipidemia	5176	(77.4)
Diabetes	1777	(26.6)

Note: Percentages may not add to 100 due to rounding.

Abbreviations: ACS = acute coronary syndrome; CABG = coronary artery bypass graft; NSTEMI = non-ST-segment elevation myocardial infarction; PCI = percutaneous coronary intervention; SD = standard deviation; STEMI = ST-segment elevation myocardial infarction; UA = unstable angina.

*ICD-9-CM Codes: Diabetes = 250.xx; Hyperlipidemia = 272.xx; Hypertension = 401.xx-4-5.xx; STEMI = 410.xx; NSTEMI = 410.7; UA = 411.1.

^†^Type of Stent determined by Diagnosis Related Group code: Drug-eluting stent = 557, 558; Bare-metal stent = 555,556; No stent = 518.

Of patients receiving DRG codes associated with PCI at index, there were 3,245 DES patients with major cardiovascular diagnosis (MCV dx, DRG 557) having a median charge of $47,510, median LOS of 3 days, and 9,313 total hospital days. In contrast, the 2,289 DES patients without MCV dx (DRG 558) had a median charge of $39,344, median LOS of 1 day, and 3,952 total hospital days. At index hospitalization, 600 BMS patients with MCV dx (DRG 555) had median charges of $40,370, median LOS of 3 days, and 1,715 total hospital days, while BMS patients without MCV dx (DRG 556) had median charges of $31,188, median LOS of 1 day, and 338 total hospital days. The 62 patients receiving no stent (DRG 518) had a median charge of $28,435, median LOS of 1 day, and 105 total hospital days. Patients with DRGs associated with PCI DES had higher total hospital days than any other DRG at index hospitalization.

**Figure 2:**
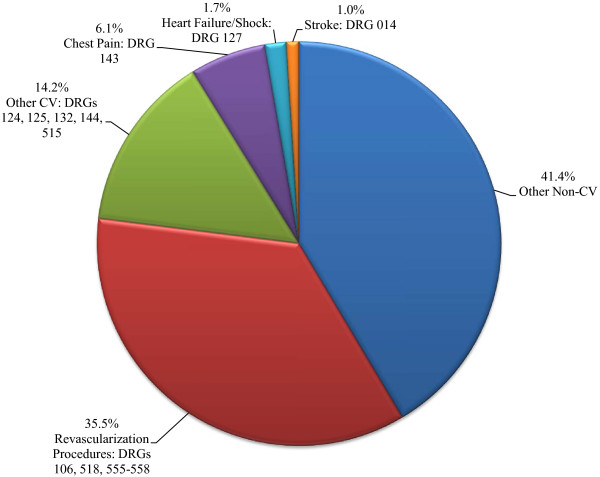
All-cause rehospitalizations by DRG code among ACS-PCI patients (n = 2,126)*. Note: Percentages may not add to 100 due to rounding. Abbreviations: ACS = acute coronary syndrome; CV = Cardiovascular; DRG = Diagnosis Related Group codes, version 24; PCI = percutaneous coronary intervention. *Rehospitalizations grouped by DRG codes.

At 30 days post-index PCI hospitalization, 6,534 (97.7%) of patients were still enrolled; however, by 15 months continuous enrollment had dropped to 4,384 (65.6%). Of the initial cohort of 6,687 commercially insured patients, 744 (11.1%) recorded an all-cause rehospitalization within 30 days post-index PCI. Of the 4,384 patients that completed the 15-month follow-up period, 2,126 (48%) patients recorded a rehospitalization for any reason, many of which were for non-CV conditions. However, 35.5% of the rehospitalizations were associated with revascularization procedures (Figure 2). Of the 744 patients who recorded an all-cause rehospitalization within 30 days post-index PCI, the majority of the rehospitalizations were for revascularization procedures 354 (47.6%), while 204 (27.4%) were for other non-CV-related rehospitalizations. At 31 days to 15 months post-index PCI, other non-CV-related rehospitalizations accounted for 48.9% of rehospitalizations compared to 29.0% for revascularization procedures. Of the 1,367 patients recording a CV-related rehospitalization, 59.3% were associated with revascularization procedures. CV-related rehospitalization frequencies are summarized in Table 2.

**Table 2:** Frequency of DRG Codes for CV-Related Rehospitalizations among ACS-PCI Patients

	**Total**	**≤30 Days post-index PCI**	**31 Days to 15 months post-index PCI**
	**(n = 1367)**	**(n = 555)**	**(n = 812)**
Revascularization Procedures	59.3%	64.9%	55.5%
Chest Pain	10.9%	9.2%	12.1%
Heart Failure/Shock	3.4%	3.1%	3.6%
Stroke	1.8%	1.8%	1.72%
Other CV	24.7%	21.1%	27.1%

Note: Percentages may not add to 100 due to rounding.

Abbreviations: ACS = acute coronary syndrome; CV = cardiovascular; DRG = Diagnosis Related Group codes version 24; PCI = percutaneous coronary intervention.

Table 3 displays median charge and total hospital days for CV-related rehospitalization episodes. In general, rehospitalizations associated with revascularization procedures costs more than those associated with chest pain, heart failure/shock, stroke, or other CV-related rehospitalizations. Among rehospitalizations, revascularization episodes associated with PCI (DRG 518, 555–558) reflected an increase in median costs, and no change in median LOS from 30 days post-index PCI to 31-days to 15-months post-index.

**Table 3:** Median Charges and Total Hospital Days for CV Rehospitalizations among ACS-PCI Patients

**DRG Group (Codes and Descriptions)**	**Within 30 Days Post-Index PCI**				**31 Day to 15 Month Post-Index PCI**			
	**Total**	**Median charge**	**Median LOS**	**Total hospital days**	**Total**	**Median charge**	**Median LOS**	**Total hospital days**
Revascularization Procedures								
106 Coronary Bypass with PTCA	9	$93266	8	70	4	$43843	6	22
518 PCI w/o stent	8	$24983	1	12	28	$28559	1	58
555 Bare metal stent with MCVdx	18	$28098	2	44	57	$45282	2	226
556 Bare metal stent w/o MCVdx	13	$23480	1	19	21	$28942	1	41
557 Drug eluting stent with MCVdx	64	$24753	2	138	95	$43879	2	205
558 Drug eluting stent w/o MCVdx	148	$34172	1	205	230	$37166	1	340
Chest Pain								
143 Chest Pain	43	$7866	1	58	118	$9270	1	167
Heart Failure/Shock								
127 Heart Failure & Shock	9	$10405	3	55	41	$13304	3	168
Stroke								
014 Intracranial hemorr & stroke w infarct	3	$11100	3	13	7	$12026	4	45
Other CV								
124 Circ disor except AMI, w cardiac cath & complex dx	21	$14613	1	38	71	$17917	2	183
125 Circ disor except AMI, w cardiac cath w/o complex dx	42	$16494	2	75	78	$17552	2	134
132 Atherosclerosis w CC	7	$10431	3	19	30	$7777	1	47
144 Oth circulatory system diagnoses w CC	11	$14584	3	44	12	$10805	3	43
515 Cardiac defibrillator impl w/o card cath	7	$120772	5	29	46	$97141	1	136

Abbreviations: ACS = acute coronary syndrome; AMI = acute myocardial infarction; CC = complications and comorbidities; CV = cardiovascular; cath = catheterization; Circ = circulatory; DRG = Diagnosis Related Group codes version 24; dx = diagnosis; hemorr = hemmorage; impl = implant; infarct = infarction; disor = disorder; LOS = length of stay; MCC = major complications and comorbidities; MCVdx = major cardiovascular diagnosis; oth = other; PCI = percutaneous coronary intervention; PTCA = Percutaneous transluminal coronary angioplasty; w = with; w/o = without.

**Figure 3:**
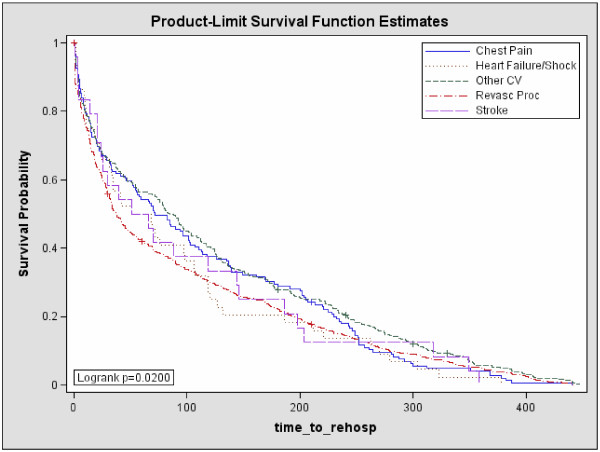
** Kaplan–Meier curves for time to CV-related rehospitalization by DRGs.** Abbreviations: CV = cardiovascular; DRGs = Diagnosis Related Groups; hosp = hospitalization; Revasc Proc = revascularization procedure.

Results for time to CV-related rehospitalization are shown in Figure 3. Patients rehospitalized for revascularization procedures had the shortest median time from post-index PCI to rehospitalization when compared to the other CV event groups. The median time to rehospitalization was 38.0 days (95% confidence interval [CI]: 33.0–45.0 days) for revascularization procedures; 59.5 days (CI: 31.0–106.0 days) for heart failure/shock, 58.5 days (CI: 24.0–144.0 days) for stroke, 72.0 days (CI: 53.0–102.0 days) for chest pain, and 85.0 days (CI: 72.0–102.0 days) for other CV-related events.

## Discussion

This study adds to the current knowledge by reporting and characterizing rehospitalizations following PCI in a commercially insured US population of patients with ACS. Patients in this analysis were typically younger than the populations reported by Curtis et al. and Jencks et al., who analyzed Medicare claims data in which patients were 65 years and older [10,11]. Our study found that of our initial study cohort, 11.1% recorded an all-cause rehospitalization within 30 days post-index PCI. This is lower than results from Curtis et al. who reported 14.6% [10]. However, an even more notable difference is that among all patients in our results from a commercially insured cohort readmitted within 30 days after the index PCI, 47.6% had a revascularization procedure compared to 27.5% in a Medicare population for the comparable 30-day period [10]. Some of this difference may be explained by the fact that both Curtis et al. and our study were unable to distinguish staged PCIs and the associated planned rehospitalizations from those that were unplanned [10]. Jencks et al. have suggested that as many as 10% of the rehospitalizations are planned, and thus these may reflect a large number of staged PCI procedures rather than true unplanned revascularizations [11]. Others have noted that PCI and other interventional procedures are performed more often on younger, less sick individuals than on older subjects, the so called “treatment risk paradox” [15]. If so, the data we report from a commercially insured population suggest that the effect size of this paradox may be much larger than expected considering randomized trials and guidelines report that the benefits of revascularization are highest among moderate and high-risk patients [16-19].

Our population is very similar to the population reported in Chastek et al., which looked at a commercially insured population between the years 2000 and 2004 [20]. While they do not report on rehospitalizations per se, in their study 24.5% of subjects experienced a subsequent ischemic event within roughly a 3-year follow-up period [20]. In our study, a total of 31.8% patients recorded a rehospitalization for all-cause over a period of up to 15 months. Chastek et al. report that the mean LOS of the index hospitalization was approximately 4.4 days, while we observed a mean LOS for index PCI of 2 to 3 days [20].

Results from this analysis are not applicable to individuals with Medicare, Medicaid, or dissimilar commercial health plans since it was focused on commercially insured patients who were primarily <65 years old. Although we intentionally did not require continuous enrollment after the index PCI, one consequence of that decision is that we do not have definitive outcomes data on approximately 1 out of 3 patients, who could have either transitioned to another health insurance plan, died, or disenrolled for other reasons. Therefore, our study was not suitable for determining overall mortality. The economic results reflect charge data reported to a managed care organization (MCO) for billing purposes and do not account for discounts or write-offs such that the charges could be converted to actual costs. The data also do not include detailed medical records and may be subject to coding and other errors in interpretation. For example, details concerning the type of PCI (primary, facilitated, or rescue) were unavailable.

## Conclusions

For ACS patients who underwent PCI, revascularization procedures represented a large portion of rehospitalizations. Revascularization procedures appear to be the most frequent, most costly, and earliest cause for rehospitalization after ACS-PCI.

## Competing interests

This study was funded by Eli Lilly and Company, Indianapolis, IN. Eric S. Meadows, PhD, Jay Bae, PhD, Anthony Zagar, MS, and Darell Heiselman, D.O. are employed by Eli Lilly and Company and own stock in Eli Lilly and Company. Tomoko Sugihara, MS, is employed by inVentiv Clinical Solutions. Krishnan Ramaswamy, PhD, is employed by Daiichi Sankyo Inc. Rebecca McCracken, MSPH, is employed by i3 Statprobe.

## Authors’ contributions

ESM was involved in the design, analysis, and interpretation of the study results. ESM revised the manuscript for important intellectual content and approved the final version. JB was involved in the design, analysis, and interpretation of the study results. JB revised the manuscript for important intellectual content and approved the final version. AZ was involved in the acquisition, analysis, and interpretation of the study results. AZ revised the manuscript for important intellectual content and approved the final version. TS was involved in the acquisition, analysis, and interpretation of the study results. TS revised the manuscript for important intellectual content and approved the final version. KR was involved in the design, analysis, and interpretation of the study results. KR revised the manuscript for important intellectual content and approved the final version. RM was involved in the analysis and interpretation of the study results. RM drafted the manuscript and approved the final version. DH was involved in the design, analysis, and interpretation of the study results. DH revised the manuscript for important intellectual content and approved the final version. All authors read and approved the final manuscript.

## Supplementary Material

Additional file 1 **Diagnosis and procedure codes.** *Diagnosis codes are from ICD-9-CM. ^†^Procedure codes are from ICD-9-CM Procedure Codes for Hospital Inpatient Services: CPT-4® Procedure Codes/HCPCS Codes for Hospital Outpatient Services and CPT-4® Codes for Physicians. (PDF 33 kb) Click here for file
