# Supplementary material for: Rehospitalization following percutaneous coronary intervention for commercially insured patients with acute coronary syndrome: a retrospective analysis
Source: BMC Res Notes. 2012 Jul 2;5:342. doi: 10.1186/1756-0500-5-342 (PMC3493265; doi:10.1186/1756-0500-5-342)
Supplement: Additional file 1 — Diagnosis and procedure codes. *Diagnosis codes are from ICD-9-CM. †Procedure codes are from ICD-9-CM Procedure Codes for Hospital Inpatient Services: CPT-4® Procedure Codes/HCPCS Codes for Hospital Outpatient Services and CPT-4® Codes for Physicians. (PDF 33 kb) [file 1756-0500-5-342-S1.pdf]

Additional File 1. Diagnosis and Procedure Codes

Diagnosis and Procedures	Code(s)
Diagnosis*	
Acute myocardial infarction	410
ST-elevation myocardial infarction	410.xx
Non-ST-elevation myocardial infarction	410.7
Unstable angina	411.1
Hemorrhage	139, 388.1, 399.8, 430-432, 456.0, 456.20, 459.0, 530.21, 530.7, 530.82, 531.0, 531.2, 531.4, 531.6, 139, 532.0, 532.2, 532.4, 532.6, 533.0, 533.2, 533.4, 533.6, 534.0, 534.2, 534.4, 534.6, 535.01, 535.11, 535.21, 535.31, 535.41, 535.51, 535.61, 535.71, 535.81, 535.91, 537.83, 537.84, 562.02, 562.03, 562.12, 562.13, 569.3, 569.85, 569.86, 578, 841, 904, 990.0-990.4, 997.02, 998.11, 998.12
Diabetes mellitus	250
Hyperlipidemia	272
Hypertension	401-405
Stroke/Transient ischemic attack	430-438
Procedure†	
Coronary artery bypass graft	361.0-361.7, 361.9, 335.10-335.14, 335.16-335.19, 335.21-335.323, 335.30, 335.33, 335.35, 335.36, 335.42, 335.72,
Percutaneous coronary intervention	00.66, 36.01, 36.02, 36.05, 36.06, 36.07, 929.73, 929.75, 929.78, 929.79-929.82, 929.84, 929.95, 929.96, G0290, G0291

*Diagnosis codes are from ICD-9-CM.

†Procedure codes are from ICD-9-CM Procedure Codes for Hospital Inpatient Services: CPT-4® Procedure Codes/HCPCS Codes for Hospital Outpatient Services and CPT-4® Codes for Physicians.
